# Research on therapeutic clinical trials including immunotherapy in triple-negative breast cancer: a bibliometric analysis

**DOI:** 10.3389/fonc.2024.1423924

**Published:** 2024-10-14

**Authors:** Qi Xu, Xiaoyu Feng, Siyuan Qin, Yu Hong, Rui Cui, Jia Liang, Zhuya Xiao, Yuan Li

**Affiliations:** ^1^ The Renmin Hospital of Wuhan University, Wuhan University, Wuhan, China; ^2^ Department of Oncology, Renmin Hospital of Wuhan University, Wuhan University, Wuhan, China

**Keywords:** bibliometric analysis, TNBC, clinical trial, neoadjuvant therapy, antibody drug conjugates, immunotherapy

## Abstract

**Background:**

Breast cancer, particularly triple-negative (TNBC), is a leading malignancy with aggressive traits and high metastasis rates. Clinical trial is an important tool for optimizing therapeutic strategies in the evaluation of the safety and efficacy for TNBC. Our bibliometric study of TNBC clinical trials aims to assess therapeutic strategies, identify trends, and explore advancements in treatment. We focus on mapping knowledge development, including key research entities and topics, and analyzing research trends and emerging methods. This analysis intends to inform future research, especially in personalized and precision medicine for TNBC.

**Methods:**

We selected publications on clinical trials for the treatment of TNBC from 1997 to 2024 in the Web of Science Core Collection (WoSCC). After an initial screening, we downloaded key data including titles, publication years, authors, countries, institutional affiliations, journals, keywords, and abstracts, and saved them in BibTex format. We then conducted a bibliometric analysis using Bibliometrix in R and VOSviewer to illustrate the prospects, highlights, and trends of TNBC treatment options. Furthermore, to emphasize the hot topics in TNBC treatment strategies, we performed a bibliometric analysis of immunotherapy using the same approach.

**Results:**

1907 publications were included, most of which were from China, Italy, and the United States. The number of annual publications has increased dramatically since 2010. The focus of TNBC clinical trial research has shifted from understanding the biology, such as breast cancer subtyping and genotyping, to novel therapeutic approaches. The major advancement in clinical trials is the switch from late-stage palliative treatment to early preoperative neoadjuvant therapy, as more TNBC cases are discovered at an early stage. Immunotherapy is also highlighted with additional alternatives for advanced or metastasized TNBC, such as targeted inhibitors with unusual mutation rates and antibody drug conjugates (ADC).

**Conclusions:**

This investigation made it apparent how immunotherapy has recently made major advancements in TNBC treatment plans and how ADCs, or targeted therapies, are currently popular for TNBC. By identifying significant papers, comprehending trending topics, and collaborating across multiple disciplines, this study may accelerate research on TNBC therapy options.

## Introduction

Nearly fifteen percent of breast cancer is triple negative breast cancer (TNBC). Compared to other subtypes, early-stage TNBC is linked to a higher risk of recurrence, and advanced stage illness has a miserable outcome with a median survival of about 18 months (mos) (1). Recent studies have shown that a certain percentage of HER2 low-expressing tumors also exist in TNBC, but for the majority of patients with TNBC do not express HER2 and also lack estrogen and progesterone receptor, thus rendering them ineffective for hormonal or anti-TNBC therapy (2). Patients with TNBC have a high recurrence rate within 3 years of diagnosis and a high mortality rate within 5 years compared to other subtypes (3). Chemotherapy is the primary strategy for systematic therapy in both the neoadjuvant and metastatic situation because there has been a dearth of specific therapies due to the lack of receptor expression (4). Nevertheless, systemic chemotherapy generates adverse effects, and the majority of patients ultimately develop resistance (5). Therefore, there is an urgent need for cutting-edge therapeutic options for TNBC that extensively improve outcomes (6).

An interdisciplinary focus entitled bibliometrics providing researchers with statistical and mathematical tools for an in-depth and dispassionate assessment of a particular field of study (7). The availability of several databases (Web of Science, Scopus, Direct Science, etc.) and software applications that facilitate the study of substantial quantities of data (VOSviewer, Leximancer, HistCite, etc.) is the reason for the popularity of bibliometric analysis (8). Thus, it has been used in various areas such as cardiovascular diseases (9), Alzheimer’s disease (10), Diabetes (11) and so on, becoming progressively more significant in evaluating hotspots and developing clinical trial procedures about TNBC treatment. Bibliometric analysis helps decipher and map cumulative scientific knowledge and evolutionary nuances in mature fields by making sense of large amounts of unstructured data in a rigorous manner (12).

Bibliometric analysis offers a new method of learning and research, allowing researchers to quickly grasp an overview and to discern the frontiers. Although some articles have employed bibliometric analysis tools, they are typically used to analyze the overall situation of TNBC research (13, 14). There has not yet been a bibliometric analysis specifically focused on clinical trials related to TNBC treatment. This study investigated the research output, the distribution of interdisciplinary research, publication sources, nations and regions with active research, institutions, and researchers, helping researchers to select appropriate publications, journals and collaborators. We also employed thematic map analysis, co-citation analysis, and keyword co-occurrence analysis to establish the conceptual framework of clinical trials focused on treating triple-negative breast cancer (TNBC). This offered us the opportunity to evaluate research frontiers, hot themes, and foundations with the goal of enlightening the current state of the field, directing future investigations, and stimulating improvements in the study of TNBC therapies. This study not only summarizes the current state of knowledge but also provides insights into future research directions, particularly in the realms of personalized and precision medicine. Furthermore, we have conducted a more in-depth analysis of the hot topic of immunotherapy and highlighted the potential of antibody-drug conjugates (ADC) as emerging treatment methods. At the same time, compared to existing research, our work offers a more macro perspective, taking into account the geographical distribution and collaboration patterns of global research, rather than just focusing on the quantity or quality of research output.

## Materials and methods

### Data sources & search strategy

**Figure 1:**
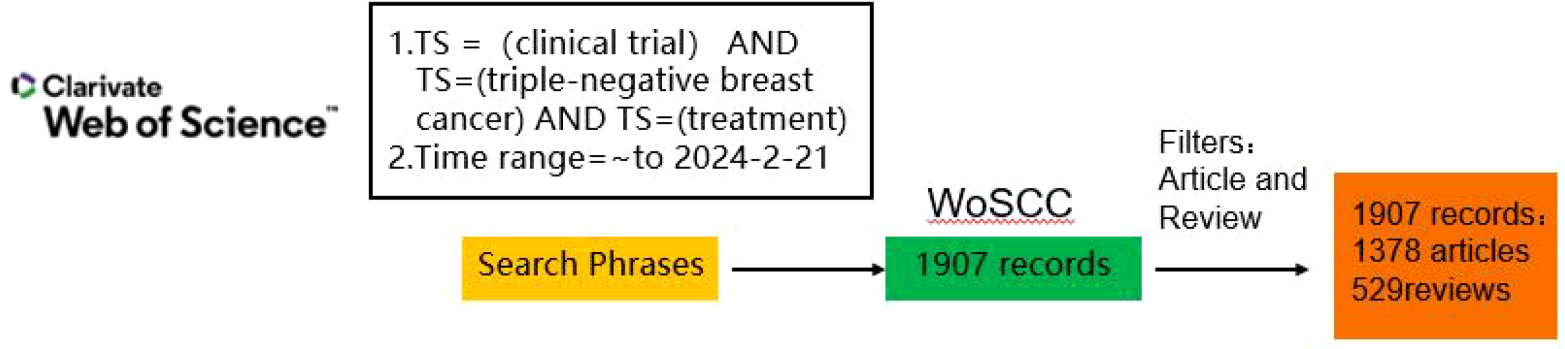
The detailed strategy of literature search presented as a schema. WoSCC, Web of Science Core Collection.

Web of Science Core Collection (WoSCC) covers journals from all disciplines and global regions, and includes only journals that demonstrate a high level of editorial rigor and best practices, as well as providing detailed metadata and citation links for each article, and containing a large number of development access articles, which can provide a wide range of bibliographic resources for bibliometric analyses, and thus be a powerful tool for bibliometric analyses. All of the material utilized in this study originates from the Science Citation Index-Expanded database from the WoSCC, and were downloaded on February 9, 2024. The search phrases were “(TS = [clinical trial]) AND TS=(triple-negative breast cancer) AND TS=(treatment)” and the keywords were “clinical trial,” “treatment,” and “triple-negative breast cancer” within WoSCC (**Figure 1**). The requirements to be included for the review articles and research about the first retrieval were limited to those published in English during 1997 and 2024. This enabled the discovery of 1907 pertinent works regarding the retravel. Each study’s critical data, containing the title, year of publication, authors, country, institutional affiliation, journal, keywords, and abstract, has been gathered in BibTex format via WoSCC and imported into RStudio for further analysis.

### Data analysis

The study gathered the metadata from databases concerning the included documents, which included active countries/regions, institutions, and researchers. The primary analysis pipelines employed R’s 3.6.3, version 3.6.3, using biblioshiny from the bibliometrix package (version 3.1.4, http://www.bibliometrix.org) (15). Bib format raw files typically were loaded; metadata (sources, writers, and citations) was subsequently calculated and plotted; this was followed by a more in-depth examination of clustering, conceptual structure, intellectual structure, and social structure. Furthermore, bibliometric analysis, which includes co-authorship analysis, keyword co-occurrence analysis, citation and co-citation analysis, and bibliographic coupling, was conducted employing VOSviewer software (version 1.6.17).

The outcomes of related functions executed by different software applications were compared in order to generate more plausible and reputable conclusions. The biblioshiny app and period co-occurrence analysis were used to generate thematic maps. The research horizons in the field of clinical trials concerning treatment for TNBC have been investigated through the use of thematic maps.

### Statistical analysis

The Spearman rank correlation coefficients were employed to assess correlations. The R software ggpubr package (version 0.4.0) developed the plots. R (version 3.6.3) was used for the statistical analysis, and all reported p-values were two-sided.

## Results

### General landscape of included documents on clinical trial about treatment in TNBC

**Figure 2:**
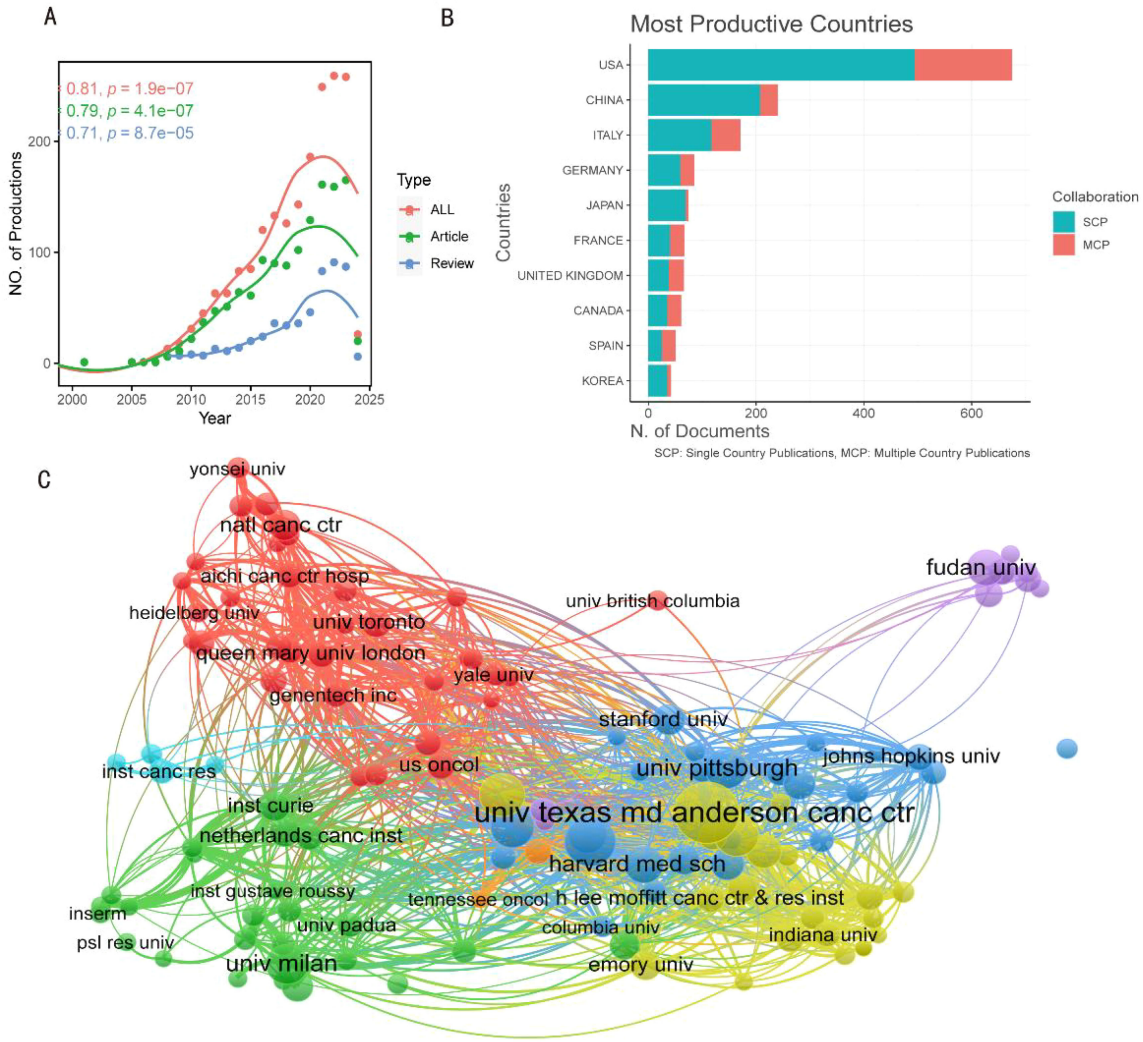
The general landscape of documents on therapeutic clinical trials about TNBC research: output, countries and affiliations. **(A)** The annual outputs of research on therapeutic clinical trials about TNBC from 1997 to 2024. **(B)** The number of documents published in different country and collaborative situation of corresponding authors. SCP, single country publications, MCP, multiple country publication. **(C)** Vos viewer-curated selective framework of scientific collaborations between affiliations from various countries. The number of publications is represented by the size of the circles, whereas different colors correspond to different clusters. The research centers’ strength of link is indicated by the thickness of the lines.

From the WoSCC database, a total of 1907 documents were gathered, all of which were unique (**Figure 1B**). WoSCC offers an extensive overview of the literature; for instance, it provides citation data, which is why files developed by WoSCC were selected as the data source for analysis. Reports from 1997 to 2010 were dispersed (**Figure 2A**). **Figure 2A** shows how the annual output of articles and reviews developed dramatically after 2010, while the total research output was not substantial before that time. 2020 marked the peak of all research creation, after which it steadily declined. A substantial association between publication year and output was identified using a polynomial model fit (Coef: 0.81, 0.79, and 0.71 for articles, reviews, and total literature, respectively) (**Figure 2A**).

**Table 1** shows that the United States (n=675) had the highest output, followed by China (n=240), Italy (n=171), Germany (n=85), and Japan (n=74), depending on the nation or location of corresponding authors. Multi-country publications (MCPs) represented 181 (26.8%) of the 675 papers from the United States. China and Italy had MCP rates of 13.8% and 31.6%, respectively, indicating that China had a lower MCP rate over Italy (**Figure 2B**, **Table 1**). The restricted cooperation between these countries or the institutions in each nation is remarkable. According to **Table 2**, the UNIV TEXAS MD ANDERSON CANC CTR (n = 340) and Fudan University (n = 77) were the two most significant affiliations between the United States and China. The University of Milan in Italy, UNIV TEXAS MD ANDERSON CANC CTR in the United States, and Fudan University in China are displayed as representative points of collaboration both within and between these nations on a collaboration map (**Figure 2C**).

**Table 1:** Most relevant countries by corresponding authors.

Country	Articles	^†^SCP	MCP	Freq	MCP_Ratio
USA	675	494	181	0.354	0.268
CHINA	240	207	33	0.126	0.138
ITALY	171	117	54	0.09	0.316
GERMANY	85	59	26	0.045	0.306
JAPAN	74	69	5	0.039	0.068
FRANCE	67	40	27	0.035	0.403
UNITED KINGDOM	66	38	28	0.035	0.424
CANADA	61	35	26	0.032	0.426
SPAIN	50	25	25	0.026	0.5
KOREA	42	35	7	0.022	0.167
AUSTRALIA	37	21	16	0.019	0.432
INDIA	33	28	5	0.017	0.152
BELGIUM	31	13	18	0.016	0.581
NETHERLANDS	28	17	11	0.015	0.393
SWEDEN	19	15	4	0.01	0.211
AUSTRIA	17	10	7	0.009	0.412
BRAZIL	16	9	7	0.008	0.438
IRELAND	16	10	6	0.008	0.375
GREECE	15	8	7	0.008	0.467
POLAND	15	12	3	0.008	0.2

^†^SCP, Single Country Publications; MCP, Multiple Country Publications.

**Table 2:** Most relevant affiliations.

Affiliation	Articles
UNIV TEXAS MD ANDERSON CANC CTR	340
FUDAN UNIV	163
DANA FARBER CANC INST	117
NETHERLANDS CANC INST	109
UNIV PITTSBURGH	91
UNIV N CAROLINA	90
MEM SLOAN KETTERING CANC CTR	86
INST CURIE	80
MAYO CLIN	78
UNIV CALIF SAN FRANCISCO	78
SUN YAT SEN UNIV	77
UNIV MILAN	73
HARVARD MED SCH	72
OHIO STATE UNIV	69
STANFORD UNIV	65
JOHNS HOPKINS UNIV	64
MED UNIV VIENNA	61
UNIV LIBRE BRUXELLES	61
UNIV WASHINGTON	61
WASHINGTON UNIV	60
UNIV COLORADO	59
CHINA MED UNIV	57
EUROPEAN INST ONCOL	56
BAYLOR COLL MED	55
NATL CANC CTR	52

**Figure 3:**
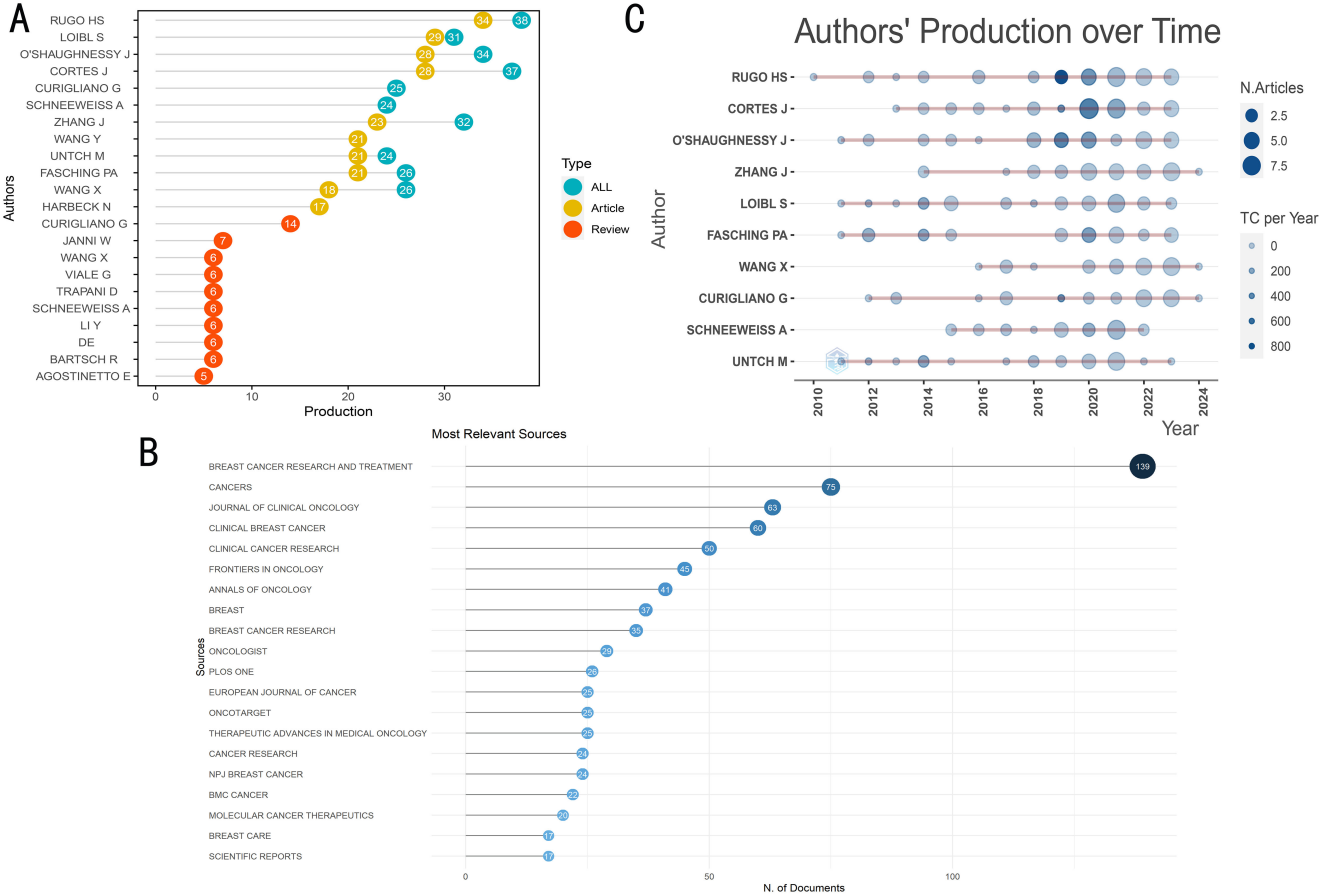
The general landscape of documents on therapeutic clinical trials about TNBC research: journals and authors. **(A)** The number of documents published in the most relevant journals. **(B)** Articles and reviews produced by highly relevant authors. **(C)** The production of top ten corresponding author over time. TC: total citations.

448 journals published the literature that was used in this investigation. The top 20 journals featuring the most documents on this keyword in cancer are listed in **Table 3**. With 75 included documents, the journal Cancers achieved second in terms of published significant publications, while the Journal of Clinical Oncology ranked third with 63 included articles (**Figure 3A**, **Table 3**). With 139 articles published, Breast Cancer Research and Treatment was the most productive journal. With 38 publications published in total, containing 34 articles and 4 reviews, RuGo HS was the most productive author (**Figure 3B**). The top 20 most relevant authors’ works over time are shown in **Figure 3C**. Additionally, there are more published articles that expand the circle. There are more citations the darker the color (**Figure 3C**).

**Table 3:** The top 20 most relevant journals in fields of therapeutic clinical trials on TNBC between 1997 and 2024.

Sources	Articles	↑JCR	IF
BREAST CANCER RESEARCH AND TREATMENT	139	Q1	3.8
CANCERS	75	Q1	5.2
JOURNAL OF CLINICAL ONCOLOGY	63	Q1	45.3
CLINICAL BREAST CANCER	60	Q2	3.1
CLINICAL CANCER RESEARCH	50	Q1	11.5
FRONTIERS IN ONCOLOGY	45	Q2	4.7
ANNALS OF ONCOLOGY	41	Q1	50.5
BREAST	37	Q1	3.1
BREAST CANCER RESEARCH	35	Q1	7.4
ONCOLOGIST	29	Q1	5.8
PLOS ONE	26	Q1	3.7
EUROPEAN JOURNAL OF CANCER	25	Q1	8.4
THERAPEUTIC ADVANCES IN MEDICAL ONCOLOGY	25	Q1	4.9
CANCER RESEARCH	24	Q1	11.2
NPJ BREAST CANCER	24	Q1	5.9
BMC CANCER	22	Q2	3.8
MOLECULAR CANCER THERAPEUTICS	20	Q1	5.7
BREAST CARE	17	Q1	2.1
SCIENTIFIC REPORTS	17	Q1	4.6
CANCER TREATMENT REVIEWS	16	Q1	11.8

↑JCR and IF were curated from Web of Science of 2024.

IF, Influence factor; JCR, Journal Citation Reports™.

### Current highlights in clinical trial about treatment in TNBC

**Figure 4:**
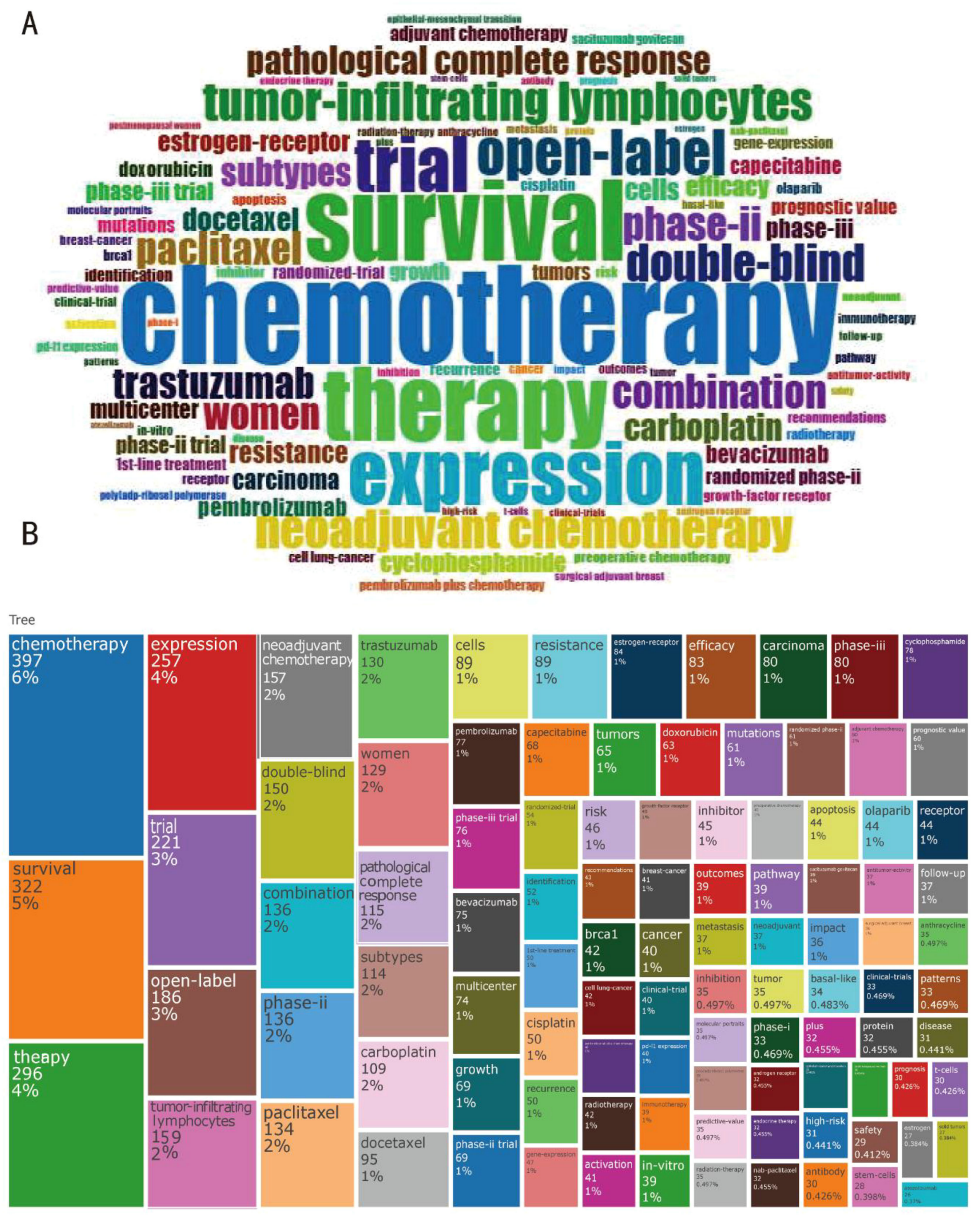
Current highlights of the field of therapeutic clinical trials about TNBC. **(A)** Word cloud plot of keywords on therapeutic clinical trials about TNBC originate from involved publications. **(B)** An analysis of the keyword tree map based on the word cloud plot.

The keywords that show up in these publications have been selected to reflect contemporary subjects that will improve scholars’ comprehension of scientific findings. These publications frequently include “clinical trial,” “treatment,” and “triple-negative breast cancer,” in addition to the terms “survivor” and “neoadjuvant chemotherapy (**Figures 4A, B**).” This indicates that paclitaxel, docetaxel, and other comparable substances are the most consistently prescribed treatments for triple-negative breast cancer. Simultaneously, there is an increasing trend in the adjuvant treatment of breast cancer utilizing monoclonal antibodies like pembrolizumab, demonstrating the significance of immunotherapy in the battle against breast cancer. Phase II trials account for the majority of the double-blind, open clinical trials for triple-negative breast cancer. These findings indicate that immunotherapy is equally important as chemotherapy in the management of breast cancer.

The 25 most cited publications worldwide are listed in **Table 4**. Goldhirsch A 2011 Annoncol had 2,728 citations, followed by Cortazar 2014 Lancet with 2,265 citations (**Table 4**). Goldhirsch a 2011 Annoncol focused on the subtype strategy of breast cancer treatment, and determined that, according to the classification of subtypes, breast cancer was suitable for endotherapy chemotherapy in along with pembrolizumab immunotherapy (16); Cortazar 2014 Lancet focuses on the neoadjuvant therapy of breast cancer (17). With all factors considered, these noteworthy studies offer a brief overview of the subject and widen experts’ viewpoints to reveal the critical role that immunotherapy plays in the treatment of breast cancer.

**Table 4:** Most globally cited documents.

Paper	DOI	Type	TC	TC per Year	Normalized TC	↑JCR	IF
GOLDHIRSCH A, 2011, ANN ONCOL	10.1093/annonc/mdr304	Article	2728	194.86	18.47	Q1	50.5
CORTAZAR P, 2014, LANCET	10.1016/S0140-6736(13)62422-8	Review	2265	205.91	24.08	Q1	168.9
ADAMS S, 2019, ANN ONCOL	10.1093/annonc/mdy517	Article	2195	365.83	27.85	Q1	50.5
ADAMS S, 2019, ANN ONCOL-a	10.1093/annonc/mdy518	Article	2195	365.83	27.85	Q1	50.5
VON MINCKWITZ G, 2012, J CLIN ONCOL	10.1200/JCO.2011.38.8595	Article	1760	135.38	18.31	Q1	45.3
NANDA R, 2016, J CLIN ONCOL	10.1200/JCO.2015.64.8931	Article	1449	161.00	23.05	Q1	45.3
SCHMID P, 2020, N ENGL J MED	10.1056/NEJMoa1910549	Article	1283	256.60	29.68	Q1	158.5
MASUDA N, 2017, N ENGL J MED	10.1056/NEJMoa1612645	Article	1017	127.13	17.97	Q1	158.5
GELMON KA, 2011, LANCET ONCOL	10.1016/S1470-2045(11)70214-5	Article	922	65.86	6.24	Q1	51.1
CORTES J, 2020, LANCET	10.1016/S0140-6736(20)32531-9	Article	831	166.20	19.22	Q1	168.9
SILVER DP, 2010, J CLIN ONCOL	10.1200/JCO.2009.22.4725	Article	732	48.80	8.51	Q1	45.3
SCHMID P, 2020, LANCET ONCOL	10.1016/S1470-2045(19)30689-8	Article	725	145.00	16.77	Q1	51.1
SIKOV WM, 2015, J CLIN ONCOL	10.1200/JCO.2014.57.0572	Article	663	66.30	11.54	Q1	45.3
O’SHAUGHNESSY J, 2011, N ENGL J MED	10.1056/NEJMoa1011418	Article	652	46.57	4.41	Q1	158.5
KIM C, 2018, CELL	10.1016/j.cell.2018.03.041	Article	586	83.71	9.74	Q1	64.5
HUDIS CA, 2011, ONCOLOGIST	10.1634/theoncologist.2011-S1-01	Review	581	41.50	3.93	Q1	5.8
TUTT A, 2018, NAT MED	10.1038/s41591-018-0009-7	Article	567	81.00	9.42	Q1	82.9
DENKERT C, 2017, LANCET	10.1016/S0140-6736(16)32454-0	Review	547	68.38	9.67	Q1	168.9
MITTENDORF EA, 2020, LANCET	10.1016/S0140-6736(20)31953-X	Article	541	108.20	12.52	Q1	168.9
VOORWERK L, 2019, NAT MED	10.1038/s41591-019-0432-4	Article	521	86.83	6.61	Q1	82

↑JCR and IF were curated from Web of Science of 2024.

IF, Influence factor; JCR, Journal Citation Reports™; TC, Total Citations; Normalized TC, calculated by dividing the actual count of citing items by the expected citation rate for documents with the same year of publication

### Trending topics of clinical trial about treatment in TNBC

**Figure 5:**
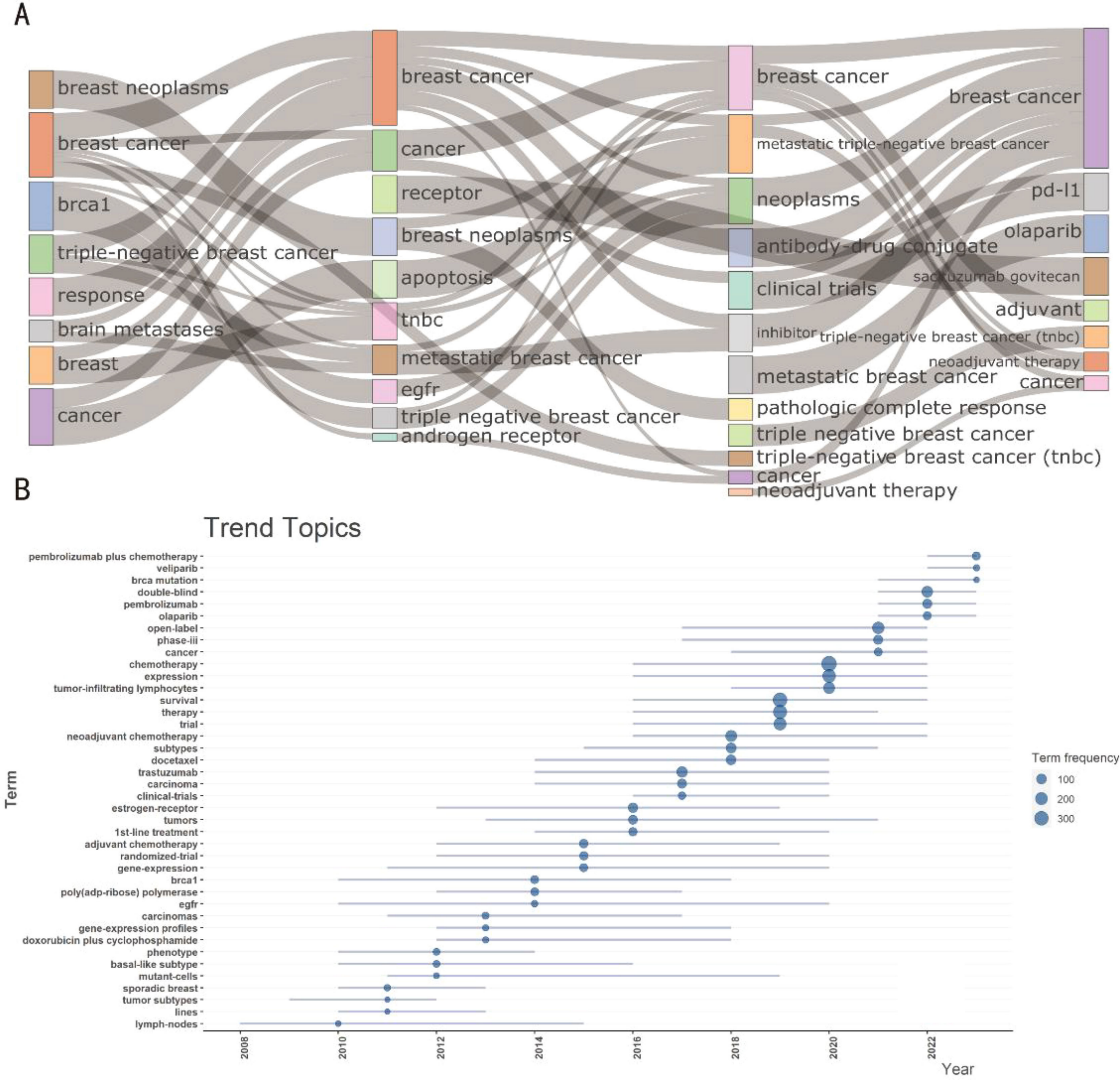
Trending topics of therapeutic clinical trials about TNBC. **(A)** Evolution of keywords across time eras. The thematic Evolution function in bibliometrix in R was used to obtain nodes and edges. The subjects of research are closely related. There were several subjects that span multiple academic disciplines and had a lot in common with one another. Concurrently, research themes changed over time and gave rise to new terminology, denoted by curving lines. **(B)** Trend topics evolution during 1997 to 2024.The size of the dot represented its frequency, while the horizon line represented its period in years.

**Figure 6:**
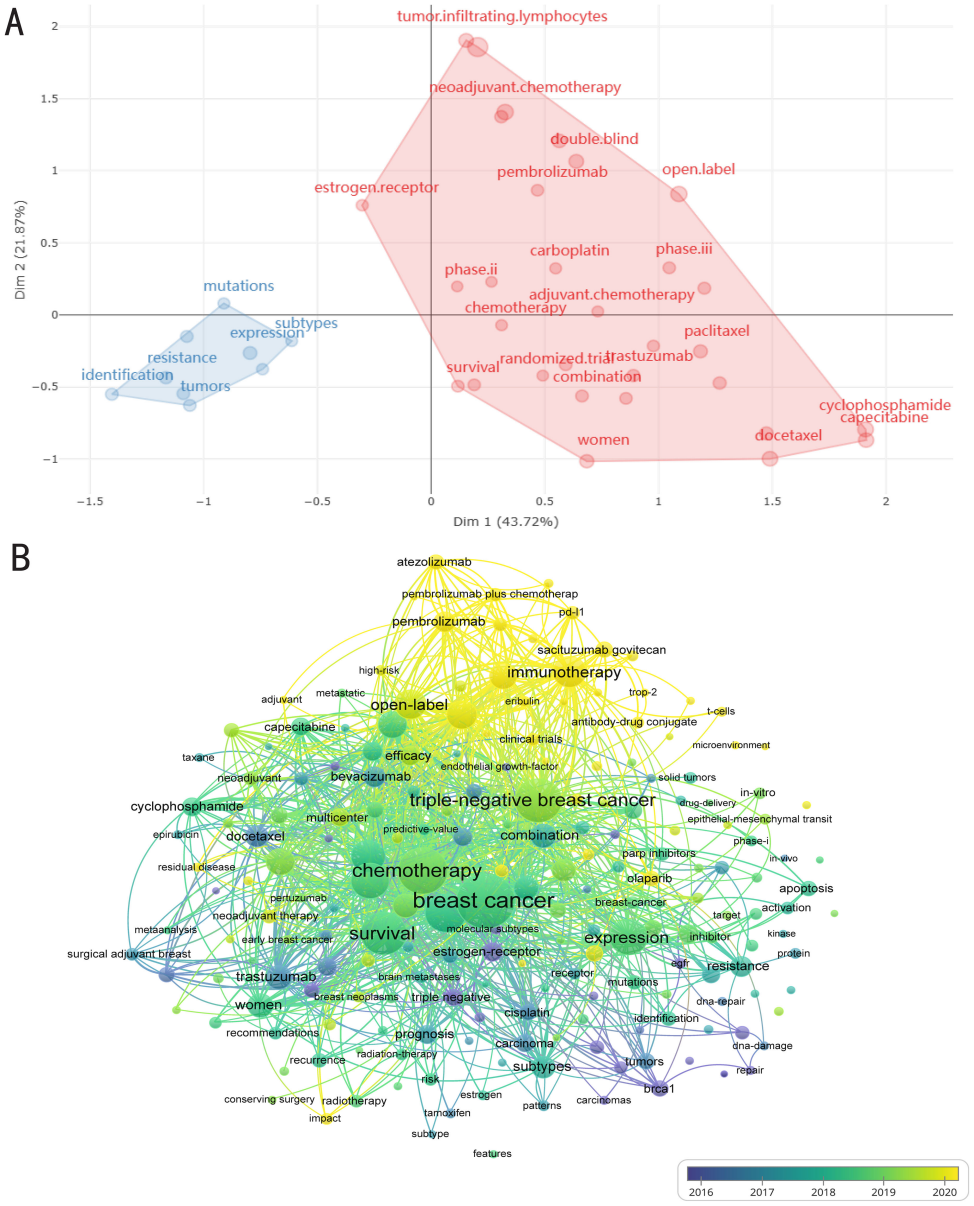
Trending topics of therapeutic clinical trials about TNBC. **(A)** Conceptual structure map of keywords. MCA, Multiple correspondence analysis. Dim: Dimension. Two colors represent two clusters. **(B)** the VOSviewer software’s examination of keyword co-occurrence. Trending topics from 2016 to 2020 are shown with lighter color for later years and darker purple for earlier years. The nodes’ sizes indicate how frequently they occur. The nodes’ appearance in the same publication is shown by the curves joining them. The likelihood of the two keywords occurring together increases with the distance between two nodes.

Hot topics of interest are presented in current publications for researchers; nevertheless, current techniques are not enough to create breakthroughs. Through keyword mutations, researchers can better grasp changes in relevant research hot topics to help obtain information more effectively. A glimpse of the evolution of keywords in recent years can be seen in **Figures 5A, B**. The image shows how a significant section of the page is devoted to numerous forms of breast cancer. Various breast cancer subtypes and metastatic tumors maintained to be hot topics from 1997 to 2014, despite negligible breakthroughs in associated therapeutic studies. Clinical trials associated with breast cancer rose after 2015, nevertheless from 2015 to 2018, genotypes and subtypes of the disease remained prominent. After 2019, the main focus of research shifted to therapeutic approaches, particularly studies concerning gene alterations associated with breast cancer, with a particular emphasis on chemotherapeutic drugs and antibody drug conjugates. Furthermore, after 2021, Pi3K inhibitors and ADC medications, sacituzumab govitecan, are becoming increasingly well-liked and could eventually replace immune profiles as the preferred option for treating TNBC (**Figures 5A, B**). As demonstrated in the **Figure 6A**, topic mapping, on the other hand, makes it possible to visualize four distinct categories of themes. Using keywords, the concept map distributes topics into four quadrants according to their centrality and density. The two main areas of keywords are research on the nature and classification of diseases as well as investigation on the effectiveness and methods of therapy. Basic subjects are represented by the bottom-right quadrant. The bottom-right quadrant displays research on triple-negative breast cancer survival rates and treatment approaches. Topics that are advancing or declining are shown in the bottom-left quadrant. Included are studies on the nature and subtypes of breast cancer, revealing a possible decline in research on the subject. It is remarkable that the “movement theme” (located in the top-right quadrant) is recognized for having a high density and centrality. Neoadjuvant therapy is a major topic in the “movement theme” further discussed in the literature. Niche topics in the top-left quadrant have limited importance in the field, although they have high density due to insignificant external links (low centrality). There are numerous topics on the development, heterogeneity, and susceptibility of cancer (**Figure 6A**).

The comprehensive findings of the VOSviewer software-assisted keyword co-occurrence analysis are presented in an organized fashion (**Figure 6B**). The most commonly searched keywords from 2016 to 2021 are displayed in light to dark purple hues. The majority of studies concerned general treatments for breast cancer, with chemotherapy and disease survival being previously identified primary themes. In addition, research on “immunotherapies,” like the PD-L1 monoclonal antibody pembrolizumab, has recently shifted from general breast cancer treatment to more specific therapies that target distinct subtypes of breast cancer, involving pembrolizumab for TNBC (**Figure 6B**). Despite the fact that immunotherapy is still quite popular right now, ADCs and other targeted treatments like sacituzumab govitecan can be noticed in the **Figure 6B**. The confidence of the results is demonstrated by the fact that these findings generally fall in line with those derived from literature-based methods (**Figure 6B**). In summary, clinical trial results indicate the critical role that immunotherapy plays in TNBC, making it a popular area of study today. These findings additionally pointed out the trend toward ADC or targeted treatments, offering fresh approaches to the management of TNBC.

### Overview of immunotherapy in TNBC

The role of immunotherapy in that leadership of TNBC is growing. We employed a similar analysis procedure using the fixed keywords “TNBC” and “immunotherapy” in order to carry out a more thorough investigation of TNBC immunotherapy (**Supplementary Figure S1A**). After 2015, this field saw quickly development, as we saw when we acquired documents from 2018 (**Supplementary Figure S1B**). With 65 papers total—55 articles and 10 reviews—the most prolific author, Li Y, has drawn attention to significant trends in the field and prompted further study (**Supplementary Figure S1C**). These articles frequently include the query words “PD-L1,” “chemotherapy,” “pembrolizumab,” and “t-cells,” in addition to the terms “immunotherapy” and “Triple-negative Breast cancer” (**Supplementary Figures S1D, E**). In the meantime, the combination of atezolizumab and albumin-bound paclitaxel was investigated in the most widely cited article by Schmid P in 2018 in N ENGL J MED. The results indicated that atezolizumab plus albumin-bound paclitaxel raised progression-free survival in the intention-to-treat population and the PD-L1-positive subgroup of patients with metastatic triple-negative breast cancer (18). Pembrolizumab monotherapy was investigated as a second-line or later therapy for previously treated mTNBC patients in the second most cited publication by Adams S in 2019 in ANN ONCOL, demonstrating its efficacy (19) (**Table S1**). Overall, improvement and assessment of immunotherapy methods that target PD-L1 are the main topics of both articles. The most cited articles’ keywords support TNBC immunotherapy’s trending challenges. A tree diagram can show particular frequencies for each keyword. Conceptual maps emphasize important research hotspots, like photodynamic therapy, and combination neoadjuvant chemotherapy and immunotherapy (**Supplementary Figures S1F, G**).

## Discussion

Bibliometric analyses are being used more and more to evaluate developments and trends in different fields of research. As for TNBC treatment, immunotherapy-such as pembrolizumab, a monoclonal antibody targeting PD-L1-is currently the hot challenge. Nevertheless, indications of ADC and other targeted therapies are already starting to appear. However, there is no study on bibliometric analysis of clinical trials regarding TNBC treatment studies. Based on available data, since 2010, there have been a significant number of clinical trial studies on TNBC treatment, with a significant upsurge in studies on TNBC immunotherapy after 2015. Research on general therapies for breast cancer was concentrated after 2010, when TNBC treatment was likely to center around chemotherapy, neoadjuvant therapy, and other methods. Furthermore, after 2020, the use of pi3K inhibitors and sacituzumab govitecan medications is starting to become obvious, indicating potential directions for TNBC treatment. However, it is also clear that performing clinical studies costs some time. The majority of pertinent journals have impact factors (IFs) that are relatively large. China comes in second to the United States in terms of research findings about TNBC immunotherapy and treatment in clinical trials. China and the United States are also actively working together on research. Another country with a high rate of research output is Italy, most likely as a result of its highly significant affiliation with the University of Milan.

It is noteworthy that the papers of Goldhirsch A., 2011, in Annals of Oncology and Cortazar, 2014, in The Lancet have received the greatest citations in clinical trials on TNBC therapy possibilities. In 2011, Goldhirsch A concentrated on subtype-specific methods to breast cancer treatment, using subtyping to figure out if the cancer is amenable to immunotherapy with pembrolizumab and chemotherapy, endocrine therapy, or chemotherapy. Cortazar, in 2014, in The Lancet, focuses on neoadjuvant therapy for breast cancer. Both articles are closely related to immunotherapy for TNBC. The two most cited investigations on the topic of immunotherapy for TNBC are by Adams S., 2019, in Annals of Oncology, and Schmid P., 2018, in The New England Journal of Medicine. In the former, atezolizumab and nab-paclitaxel are combined, and it is shown that this prolongs progression-free survival in patients with metastatic triple-negative breast cancer who have intention-to-treat as well as in the subgroup of patients who have PD-L1 positive. The latter investigated pembrolizumab monotherapy as a follow-up or second-line treatment for patients with mTNBC who had already received treatment. Studies performed in both directions emphasize the role immunotherapy plays in TNBC, with pembrolizumab serving as the main PD-L1 monoclonal antibody medication.

Immunotherapy for TNBC is growing more and more popular, according to two levels of analysis: the topic maps of the literature studies and the keyword co-occurrence analysis in the VOSviewer software. Chemotherapy and neoadjuvant therapy, the typical treatment for breast cancer, were the topic of previous study. But these days, immunotherapies like “pembrolizumab” and “PD-L1 monoclonal antibody”—targeted medicines for various subtypes of breast cancer—have acquired popularity as a means of treating TNBC, and their significance cannot be ignored. Though immunotherapy is now quite popular, alternative targeted medicines, like ADC medications and Pi3K inhibitors, are starting to gain traction and could end up becoming the standard for treating TNBC in the future.

Due to its heterogeneity and acquired resistance, single-agent therapy has not produced encouraging outcomes in clinical studies battling invasive TNBC, although demonstrating promising results in cell line and preclinical models. Presently, about eighty percent of clinical studies are looking into novel combination therapy-based therapies for TNBC (20).

Chemotherapy or neoadjuvant chemotherapy alone is not the best option for treating TNBC. Chemotherapy is not sufficient to treat TNBC; more sophisticated targeted therapies are required to enhance the prognosis of particular patient subgroups (21). TNBC is a heterogeneous tumor with higher genetic instability, frequent copy number changes, and complex structural rearrangements, and these features make TNBC more suitable for immunotherapy. Currently, immune checkpoint inhibitors combined with chemotherapy, PARP inhibitors, cancer vaccines, or NK cell treatment are the main targets of immunotherapy research for TNBC (22). PD-L1 is an attractive target for therapy because of its high mutation activity and 20% elevation in TNBC patients (23). However, the discrepancy between immunohistochemical PD-L1 assay performance and inter-reader reproducibility has also raised concerns. Related studies have shown that high tumor-infiltrating lymphocytes (TILs) are also associated with response to PD-1/PD-L1 inhibitors in breast cancer patients. Thus, the combined analysis of PD-L1 and TILs could serve as a more comprehensive immuno-oncology biomarker (24). Further published studies may soon allow for a more thorough bibliometric analysis on the application of PD-L1 in immunotherapy for TNBC.

Targeted therapy has become increasingly important in TNBC, providing new options for TNBC treatment through the discovery of new therapeutic targets. Targeted therapy for TNBC is mainly based on different subtypes of gene expression profile and related molecular pathways. TNBC can currently be further categorized into subtypes, with different treatment strategies chosen according to the subtype. Basal-like TNBC (BL-TNBC) can be subdivided into two subgroups, the BL1 and BL2 subgroup. BL1-TNBC is similar to that seen in BRCA1 or BRCA2 mutation carriers (25). Tumors in this group tend to have a high genomic instability, high mutational burden, and deficiencies in homologous recombination repair (HRR). They may also express certain markers such as cytokeratins 5/6 (CK5/6) and epidermal growth factor receptor (EGFR), and lack the expression of HER2 (26). Tumors in the BL2 group may not exhibit the same level of genomic instability or HRR deficiency. They might still express basal cytokeratins and EGFR but could have different responses to treatment and outcomes compared to the BL1 group. Thus, targeting DNA repair defects can be an effective therapeutic strategy for TNBC of the BL1 subgroup characterized by BACRness or BRCA mutations, and platinum and PARP inhibitors have been shown to have significant efficacy in the treatment of such TNBC (27). Moreover, mesenchymal-like TNBC are represented by enriched genes involved in the biological regulation of EMT and CSC, and thus, therapeutic strategies such as c-MET-targeted therapy, immune checkpoint blockade, and so on, are effective. For HER2-enriched TNBC, EGFR-targeted therapy and PI3K/AKT/mTOR pathway-targeted therapy are effective (28). As a result, subtype-based therapeutic strategies are being investigated. Relevant studies have shown that AR targeted therapy is typically used to treat the LAR subtype; PD-1/PD-L1 inhibitors are usually prescribed to treat the IM subtype; PARP inhibitors are typically used to treat the BL1 subtype; mTOR inhibitors are typically used to treat the BL2 subtype; and PI3K inhibitors are typically used to treat the M/MSL subtype (29, 30). Relevant clinical trials have demonstrated that AKT inhibitors such as capivasertib and ipatasertib play a significant role in the PI3K/AKT/mTOR signaling pathway, both occupying important positions in the treatment of metastatic triple-negative breast cancer (TNBC) (31, 32). However, the therapeutic effects of the related treatments in different TNBC subtypes are still under investigation, and targeted therapies are no longer limited to specific subtypes. The percentage of various therapies can be inferred from the frequency with which these terms occur. Nonetheless, TNBC treatment remains challenging particularly because other breast tumors do not express similar therapeutic targets. Another problem with TNBC is its heterogeneity; different subtypes of triple-negative cancers respond variably to the therapies that are now available (33).

Although immunotherapy has been a well-liked addition to TNBC’s expanding toolkit, only a small percentage of patients have shown any real improvement. Novel immune checkpoint targets, intratumoral injections that directly modify the tumor microenvironment, and small molecule inhibitors are among the latest strategies being researched in an effort to elicit immune responses in refractory cancers. Given the risk for irreversible autoimmune damage and the lack of predictive accuracy of early PD-L1 expression, it is vital to identify patients who will benefit most from treatment as the use of immunotherapy to treat early-stage TNBC increases (1). Antibody-drug conjugates, or ADCs, have shown potential as a practical and effective class of cancer treatments during recent trials. Trastuzumab deruxtecan was the first ADC medication authorized for the treatment of TNBC, while sacituzumab govitecan came next. Even though Trastuzumab deruxtecan works by targeting binding to the receptor HER2, it also has an important role in TNBC. On the one hand, TNBC usually do not express HER2, but in some cases do express low to moderate levels of HER2. HER2 status is based on the results of HER2 testing, called an immunohistochemistry (IHC) test or fluorescence *in situ* hybridization (FISH) test. HER2-positive is defined as IHC 3+ or FISH-positive. Therefore, certain TNBCs may have low or moderate expression of HER2 (IHC 1+ or 2+). Moreover, therapeutics may have a bystander effect, whereby the cytotoxicity released kills tumor cells that do not express ADC-targeted antigens cells, thus making them an effective means of treating TNBC (34). The FDA has approved the most recent class of antibody-drug coupling payloads, which is represented by these medications taken together. However, as studies continue to progress, it has been found that only a fraction of TNBC patients respond to ADC therapy and often develop resistance. Therefore, studies on combination therapy are becoming more and more essential (35).

Our study also has certain limitations. On the one hand, the limitations of our study are reflected in potential biases within the analysis process, including sample selection bias, the limitations of the data collection time frame, and possible flaws in the analytical methods (36). On the other hand, although our study captures the shifts in research hotspots within the field of TNBC treatment, it does not provide specific details about related studies. Different from traditional review, bibliometric analysis cannot offer systematic details; researchers still need to read specific documents. Future perspectives need professional instinct and accumulated experience, due to lower weight among all the papers which may be overshadowed by highly influential publications.

Our study employs a methodological approach that swiftly amalgamates the insights derived from literature analysis. Our synthesis reveals that the cutting-edge advancements in TNBC clinical trials are predominantly centered around antibody-drug conjugates (ADCs) and targeted therapies. This trend underscores a blurring of the traditional lines that define breast cancer subtypes, suggesting that certain individuals with triple-negative breast cancer may derive benefits from therapies involving HER2-targeting ADCs (37). Significantly, the potential for groundbreaking progress in this domain is increasingly linked to the emergence of novel technological advancements. By conducting a bibliometric analysis keyed to the utilization of ADCs in TNBC, we can deduce that the advent of innovative ADC technologies may precipitate a transformative shift in the field. Notably, the prospect of ADCs incorporating radioactive isotopes holds the promise of enhancing the therapeutic impact of radiation treatments for patients with advanced metastatic triple-negative disease (38). Moreover, the advent of therapies that target less common molecular markers has effectively surmounted the constraints imposed by conventional subtyping, heralding an era where classification based on specific molecular targets is poised to become a pivotal direction in the evolution of cancer treatment strategies (39).

## Data Availability

The original contributions presented in the study are included in the article/**Supplementary Material**. Further inquiries can be directed to the corresponding authors.
