# Supplementary material for: Research on therapeutic clinical trials including immunotherapy in triple-negative breast cancer: a bibliometric analysis
Source: Front Oncol. 2024 Oct 14;14:1423924. doi: 10.3389/fonc.2024.1423924 (PMC11513593; doi:10.3389/fonc.2024.1423924)
Supplement: Supplementary file 1 [file Presentation1.pdf]

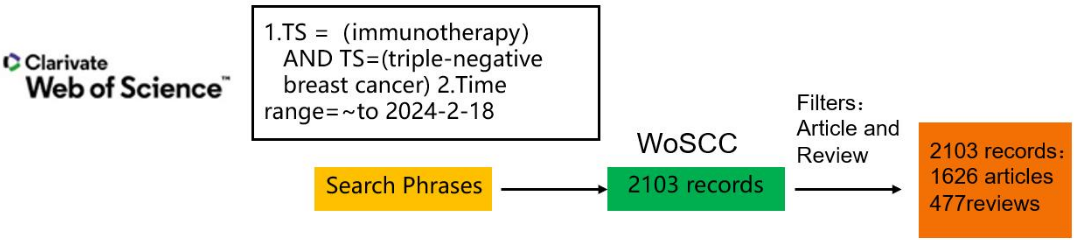

Figure S1A. The detailed strategy of literature search presented as a schema. WoSCC: Web of Science Core Collection.

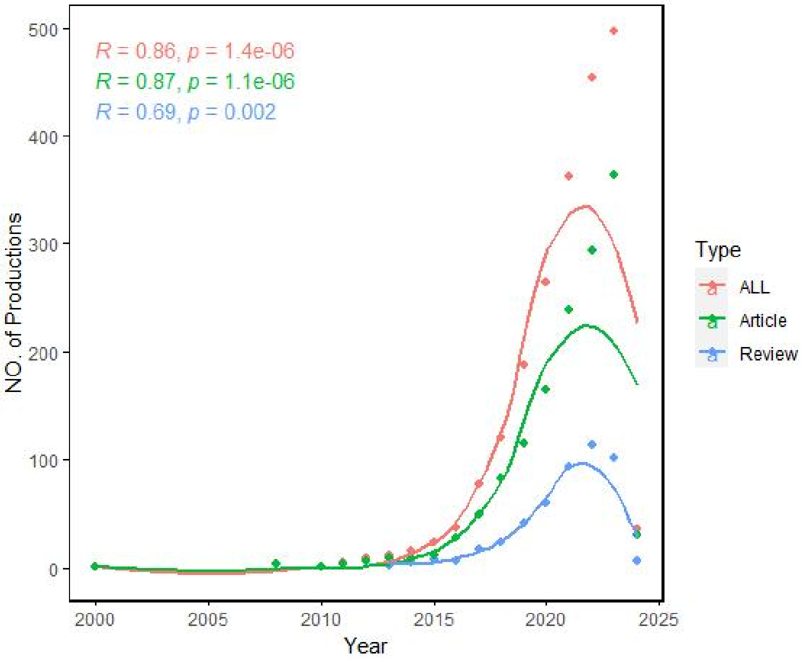

Figure S1B. The number of documents published in different country and collaborative situation of corresponding authors. SCP: single country publications.

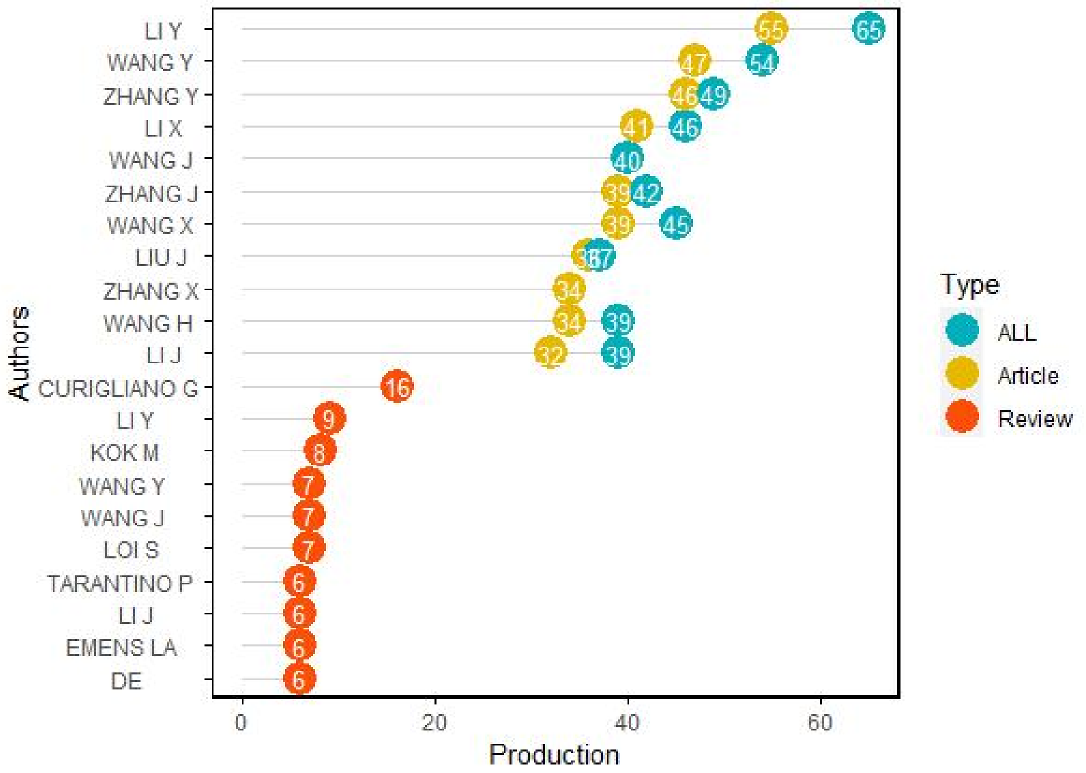

Figure S1C. Articles and reviews produced by highly relevant authors.

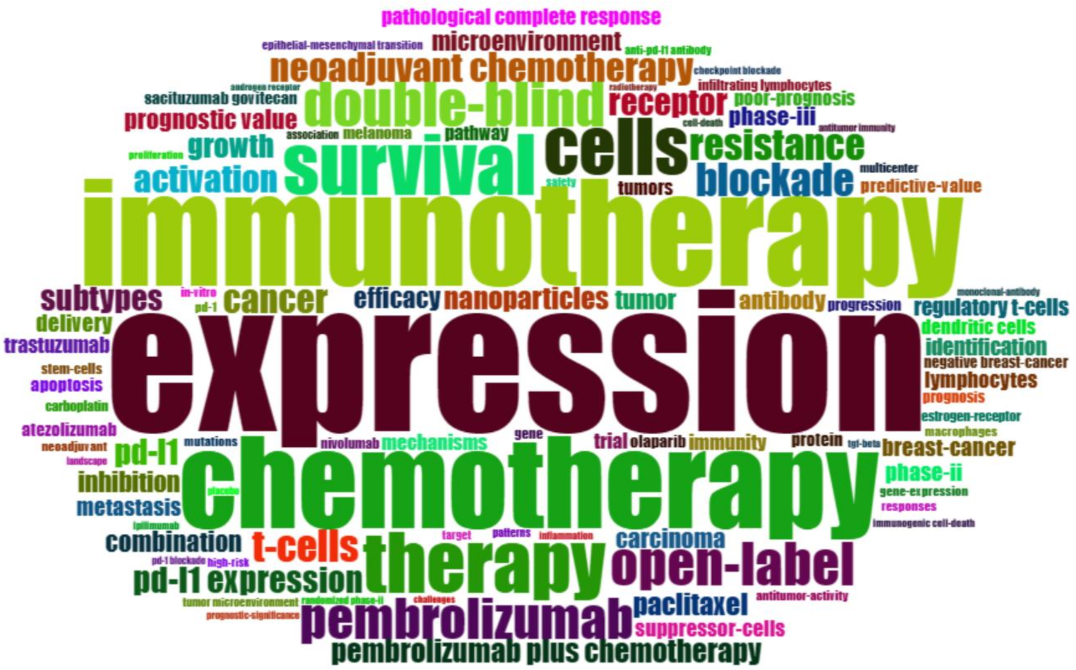

Figure S1D. Word cloud plot of keywords on TNBC immunotherapy originate from involved publications.

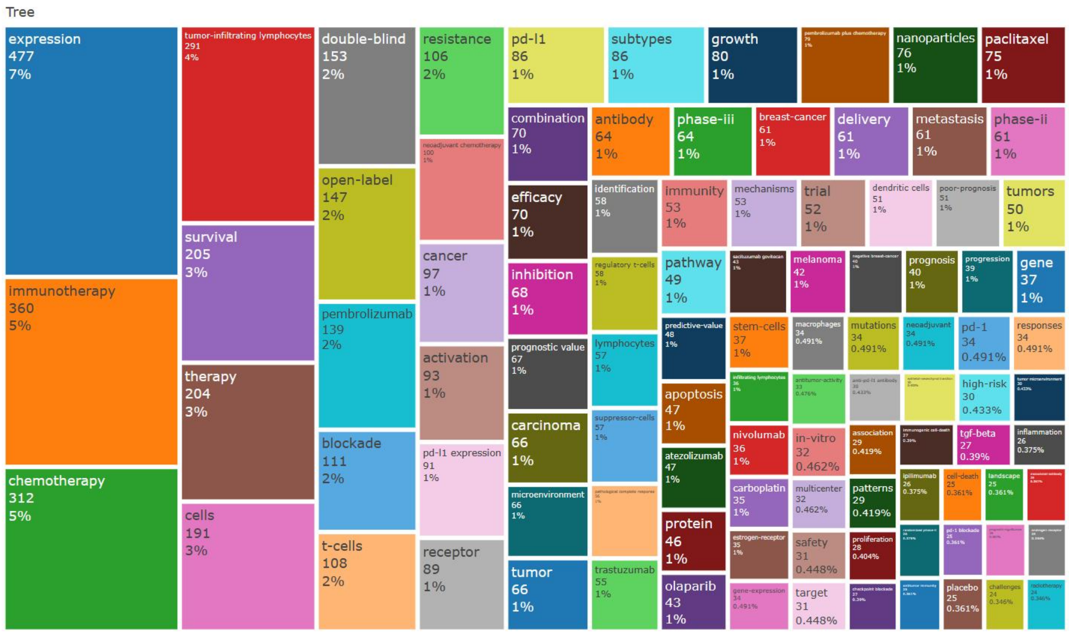

Figure S1E. An analysis of the keyword tree map based on the word cloud plot.

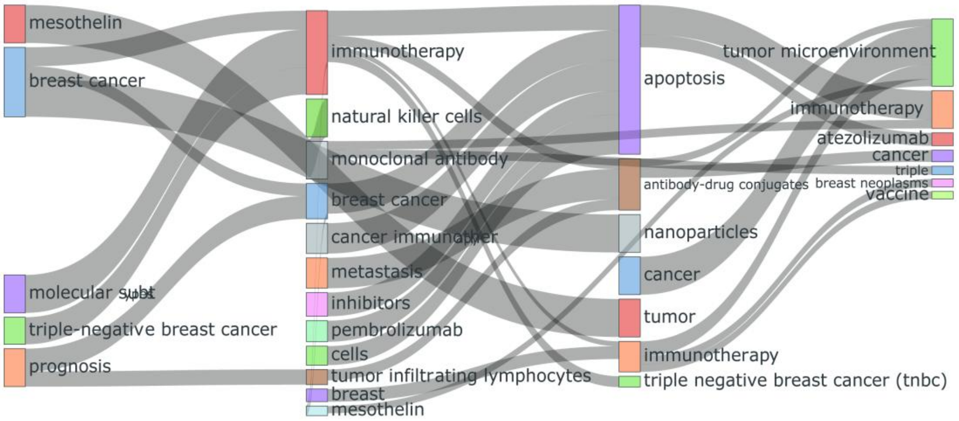

Figure S1F. Evolution of keywords across time eras. The thematic Evolution function in bibliometrix in R was used to obtain nodes and edges. The subjects of research are closely related. There were several subjects that span multiple academic disciplines and had a lot in common with one another. Concurrently, research themes changed over time and gave rise to new terminology, denoted by curving lines.

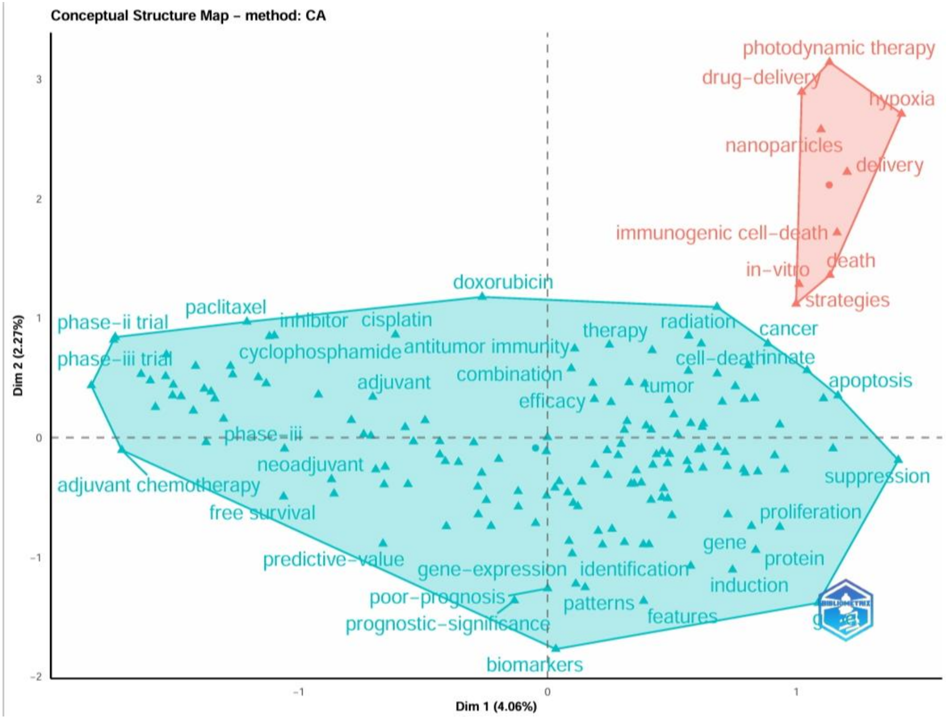

Figure S1G. Conceptual structure map of keywords. MCA: Multiple correspondence analysis.

Dim: Dimension. Two colors represent two clusters.

Table S1. Most globally cited documents.

Paper	DOI	Type	TC	TC per Year	Normalized TC	JCR	IF
SCHMID P, 2018, N ENGL J MED	10.1056/NEJMoa1809615	Article	2597	371.00	35.49	Q1	158.5
ADAMS S, 2019, ANN ONCOL	10.1093/annonc/mdy517	Article	2164	360.67	28.41	Q1	50.5
ADAMS S, 2019, ANN ONCOL-a	10.1093/annonc/mdy518	Article	2164	360.67	28.41	Q1	50.5
BIANCHINI G, 2016, NAT REV CLIN ONCOL	10.1038/nrclinonc.2016.66	Review	1634	181.56	10.80	Q1	78.8
NANDA R, 2016, J CLIN ONCOL	10.1200/JCO.2015.64.8931	Article	1440	160.00	9.52	Q1	45.3
SCHMID P, 2020, N ENGL J MED	10.1056/NEJMoa1910549	Article	1248	249.60	35.64	Q1	158.5
LOIBL S, 2021, LANCET	10.1016/S0140-6736(20)32381-3	Review	756	189.00	36.01	Q1	168.9
SCHMID P, 2020, LANCET ONCOL	10.1016/S1470-2045(19)30689-8	Article	718	143.60	20.50	Q1	51.1
BARKAL AA, 2019, NATURE	10.1038/s41586-019-1456-0	Article	600	100.00	7.88	Q1	64.8
EMENS LA, 2019, JAMA ONCOL	10.1001/jamaoncol.2018.4224	Article	499	83.17	6.55	Q1	28.4
EMENS LA, 2018, CLIN CANCER RES	10.1158/1078-0432.CCR-16-3001	Review	482	68.86	6.59	Q1	11.5
DAVIS AA, 2019, J IMMUNOTHER CANCER	10.1186/s40425-019-0768-9	Article	478	79.67	6.27	Q1	10.9
STANTON SE, 2016, J IMMUNOTHER CANCER	10.1186/s40425-016-0165-6	Review	463	51.44	3.06	Q1	10.9
LOI S, 2016, CLIN CANCER RES	10.1158/1078-0432.CCR-15-1125	Article	384	42.67	2.54	Q1	11.5
WANG C, 2017, NAT BIOMED ENG	10.1038/s41551-016-0011	Article	380	47.50	7.75	Q1	28.1
LOIBL S, 2019, ANN ONCOL	10.1093/annonc/mdz158	Article	379	63.17	4.97	Q1	50.5
LOI S, 2013, PROC NATL ACAD SCI U S A	10.1073/pnas.1222251110	Article	372	31.00	3.99	Q1	11.1
PANTELIDOU C, 2019, CANCER DISCOV	10.1158/2159-8290.CD-18-1218	Article	370	61.67	4.86	Q1	28.2
CHEN YT, 2011, PLOS ONE	10.1371/journal.pone.0017876	Article	338	24.14	3.14	Q2	3.7
LI CW, 2018, CANCER CELL	10.1016/j.ccell.2018.01.009	Article	336	48.00	4.59	Q1	50.3

↑ JCR and IF were curated from Web of Science of 2024.

IF: Influence factor; JCR: Journal Citation Reports™; TC: Total Citations; Normalized TC: calculated by dividing the actual count of citing items by the expected citation rate for documents with the same year of publication.
