# Supplementary material for: Research on therapeutic clinical trials including immunotherapy in triple-negative breast cancer: a bibliometric analysis
Source: Front Oncol. 2024 Oct 14;14:1423924. doi: 10.3389/fonc.2024.1423924 (PMC11513593; doi:10.3389/fonc.2024.1423924)
Supplement: Supplementary file 2 [file Table1.pdf]

Paper	DOI	Type	TC	TC per Year	Normalized TC	JCR	IF
SCHMID P, 2018, N ENGL J MED	10.1056/NEJMoa1809615	Article	2597	371.00	35.49	Q1	158.5
ADAMS S, 2019, ANN ONCOL	10.1093/annonc/mdy517	Article	2164	360.67	28.41	Q1	50.5
ADAMS S, 2019, ANN ONCOL-a	10.1093/annonc/mdy518	Article	2164	360.67	28.41	Q1	50.5
BIANCHINI G, 2016, NAT REV CLIN ONCOL	10.1038/nrclinonc.2016.66	Review	1634	181.56	10.80	Q1	78.8
NANDA R, 2016, J CLIN ONCOL	10.1200/JCO.2015.64.8931	Article	1440	160.00	9.52	Q1	45.3
SCHMID P, 2020, N ENGL J MED	10.1056/NEJMoa1910549	Article	1248	249.60	35.64	Q1	158.5
LOIBL S, 2021, LANCET	10.1016/S0140-6736(20)32381-3	Review	756	189.00	36.01	Q1	168.9
SCHMID P, 2020, LANCET ONCOL	10.1016/S1470-2045(19)30689-8	Article	718	143.60	20.50	Q1	51.1
BARKAL AA, 2019, NATURE	10.1038/s41586-019-1456-0	Article	600	100.00	7.88	Q1	64.8
EMENS LA, 2019, JAMA ONCOL	10.1001/jamaoncol.2018.4224	Article	499	83.17	6.55	Q1	28.4
EMENS LA, 2018, CLIN CANCER RES	10.1158/1078-0432.CCR-16-3001	Review	482	68.86	6.59	Q1	11.5
DAVIS AA, 2019, J IMMUNOTHER CANCER	10.1186/s40425-019-0768-9	Article	478	79.67	6.27	Q1	10.9
STANTON SE, 2016, J IMMUNOTHER CANCER	10.1186/s40425-016-0165-6	Review	463	51.44	3.06	Q1	10.9
LOI S, 2016, CLIN CANCER RES	10.1158/1078-0432.CCR-15-1125	Article	384	42.67	2.54	Q1	11.5
WANG C, 2017, NAT BIOMED ENG	10.1038/s41551-016-0011	Article	380	47.50	7.75	Q1	28.1
LOIBL S, 2019, ANN ONCOL	10.1093/annonc/mdz158	Article	379	63.17	4.97	Q1	50.5
LOI S, 2013, PROC NATL ACAD SCI U S A	10.1073/pnas.1222251110	Article	372	31.00	3.99	Q1	11.1
PANTELIDOU C, 2019, CANCER DISCOV	10.1158/2159-8290.CD-18-1218	Article	370	61.67	4.86	Q1	28.2
CHEN YT, 2011, PLOS ONE	10.1371/journal.pone.0017876	Article	338	24.14	3.14	Q2	3.7
LI CW, 2018, CANCER CELL	10.1016/j.ccell.2018.01.009	Article	336	48.00	4.59	Q1	50.3
